# Effects of Metabolic Syndrome on Semen Quality and Circulating Sex Hormones: A Systematic Review and Meta-Analysis

**DOI:** 10.3389/fendo.2020.00428

**Published:** 2020-08-11

**Authors:** Liming Zhao, Aixia Pang

**Affiliations:** ^1^Department of Nuclear Medicine, Linyi People's Hospital, Linyi, China; ^2^Department of Urology, Linyi People's Hospital, Linyi, China

**Keywords:** metabolic syndrome, semen quality, sex hormones, meta-analysis, male infertility

## Abstract

Previous studies were controversial in the effects of metabolic syndrome (MetS) on semen quality and circulating sex hormones, and thus we conducted a systematic review and meta-analysis to clarify the association. A systematic search was conducted in public databases to identify all relevant studies, and study-specific standardized mean differences (SMD) and 95% confidence intervals (CI) were pooled using a random-effects model. Finally, 11 studies were identified with a total of 1,731 MetS cases and 11,740 controls. Compared with the controls, MetS cases had a statistically significant decrease of sperm total count (SMD: −0.96, 95% CI: −1.58 to −0.31), sperm concentration (SMD: −1.13, 95% CI: −1.85 to −0.41), sperm normal morphology (SMD: −0.61, 95% CI: −1.01 to −0.21), sperm progressive motility (SMD: −0.58, 95% CI: −1.00 to −0.17), sperm vitality (SMD: −0.83, 95% CI: −1.11 to −0.54), circulating follicle-stimulating hormone (SMD: −0.87, 95% CI: −1.53 to −0.21), testosterone (SMD: −5.61, 95% CI: −10.90 to −0.31), and inhibin B (SMD: −2.42, 95% CI: −4.52 to −0.32), and a statistically significant increase of sperm DNA fragmentation (SMD: 0.76, 95% CI: 0.45 to 1.06) and mitochondrial membrane potential (SMD: 0.89, 95% CI: 0.49 to 1.28). No significant difference was found in semen volume, sperm total motility, circulating luteinizing hormone (LH), estradiol, prolactin and anti-Müllerian hormone (AMH) (*P* > 0.05). In conclusion, this meta-analysis demonstrated the effects of MetS on almost all the semen parameters and part of the circulating sex hormones, and MetS tended to be a risk factor for male infertility. Further larger-scale prospective designed studies were needed to confirm our findings.

## Introduction

Metabolic syndrome (MetS) is a complex of clinical conditions characterized by abdominal obesity, dyslipidemia, hypertension and insulin resistance (1, 2). Despite a link between MetS and poor health status, its impact on male infertility is still under discussion (3). First, as an important feature of MetS, obesity was found to reduce semen quality by altering the sex hormone levels and semen microenvironment, and inducing oxidative stress damage in sperms and interstitial glands (4, 5). However, the meta-analysis by MacDonald et al. (6) found no significant association between body mass index (BMI) and semen parameters, while it was associated with the prevalence of azoospermia or oligozoospermia in the meta-analysis of Sermondade et al. (7). Second, dyslipidemia, hypertension, and diabetes mellitus could also affect male reproductivity by decreasing testosterone secretion and causing testicular damage and erectile dysfunction (8–10).

As a collection of these features, MetS was thought to be involved in the pathogenesis of male infertility. The meta-analysis by Brand et al. (11) indicated a lower level of testosterone in men with MetS, but it failed to evaluate the effects of MetS on other circulating sex hormones and semen quality. The review by Corona et al. (12) analyzed the relationship between obesity, its metabolic complications and male hypogonadism (HG), and their contribution to the pathogenesis of erectile dysfunction. The meta-analysis by Rastrelli et al. (13) evaluated the association between MetS and HG, the association between HG and specific MetS components (central obesity, glucose tolerance, dyslipidemia, and hypertension), the association between MetS and sexual symptoms, the effects of MetS treatment on HG, and the effects of HG treatment on MetS. However, no meta-analyses have systematically and quantitatively evaluated the effects of MetS on both semen quality and several sex hormones in men, although there existed obvious controversies in original studies. Thus, this meta-analysis aimed to clarify the role of MetS in male infertility by assessing its impact on both semen and hormonal parameters.

## Materials and Methods

### Search Strategy

The databases of PubMed and Embase were searched for relevant studies published up to March 1st, 2020, using the key words: (“metabolic syndrome” OR “syndrome X” OR “insulin resistance syndrome” OR “metabolic X syndrome” OR “dysmetabolic syndrome” OR “reaven syndrome” OR “metabolic cardiovascular syndrome”) AND (“sperm” OR “semen” OR “spermatozoa” OR “sperm count” OR “sperm concentration” OR “semen quality” OR “semen parameters” OR “sperm quantity” OR “total sperm count” OR “azoospermia” OR “oligozoospermia”). We also reviewed the references of related studies and reviews for undetected studies. This study was approved by the ethics committee of Linyi People's Hospital (No. 2020006).

### Study Selection and Exclusion

Two authors (LZ and AP) reviewed the studies independently. The inclusion criteria were as follows: (i) focused on MetS cases and controls; (ii) any measurement of semen volume, total sperm count, sperm concentration, sperm normal morphology, sperm total motility, sperm progressive motility, sperm vitality, sperm DNA fragmentation or mitochondrial membrane potential (MMP); (iii) measurement levels were presented as mean or median with standard error (SD), 95% confidence interval (95% CI), range or inter-quartile range (IQR). The exclusion criteria were as follows: abstracts without full texts, reviews, case reports, animal studies, and studies in languages other than English.

### Data Extraction and Quality Assessment

Two authors (LZ and AP) extracted the data by a standardized collection form. All differences were resolved by discussion. In each study, the following information was extracted: first author, publication year, study area, study population, diagnosis criteria of MetS, semen or sex hormone parameters, sample size per group, and the testing values of semen parameters and circulating sex hormones. For studies from the same area, we also reviewed the medical center and study time to exclude duplicate publications. The Newcastle-Ottawa Scale (NOS) was used to assess the methodological quality of included studies (http://www.ohri.ca/programs/clinical_epidemiology/oxford.asp).

### Statistical Analysis

If the measurement levels were presented as mean or median with range or IQR, they were converted to mean ± SD according to the methods by Wan et al. (14). Study-specific standardized mean differences (SMD) and the corresponding 95% CI were pooled by the Inverse Variance method to evaluate the effects of MetS on selected parameters. A random-effects model was used as the pooling method, which considered both within-study and between-study variation. The heterogeneity among studies was estimated by *Q* test and *I*^2^ statistic, and *I*^2^ > 50% represented substantial heterogeneity (15). Egger's test was used to detect publication bias (16). Sensitivity analysis was conducted to estimate the stability of the meta-analysis by omitting one study at a time during repeated analyses. Subgroup analysis was conducted on the study cohorts [including fertile cohort, infertile cohort, and the general cohort (not specified)], ethnicity and study area (from developed or developing area) to evaluate the effects of potential confounding factors on the primary results. Statistical analyses were performed using Review Manager 5.3 (RevMan, The Nordic Cochrane Center, The Cochrane Collaboration), and Egger's test was realized with software STATA version 12.0 (StataCorp LP, College Station). *P* < 0.05 was considered statistically significant.

## Results

### Characteristics of Included Studies

**Figure 1:**
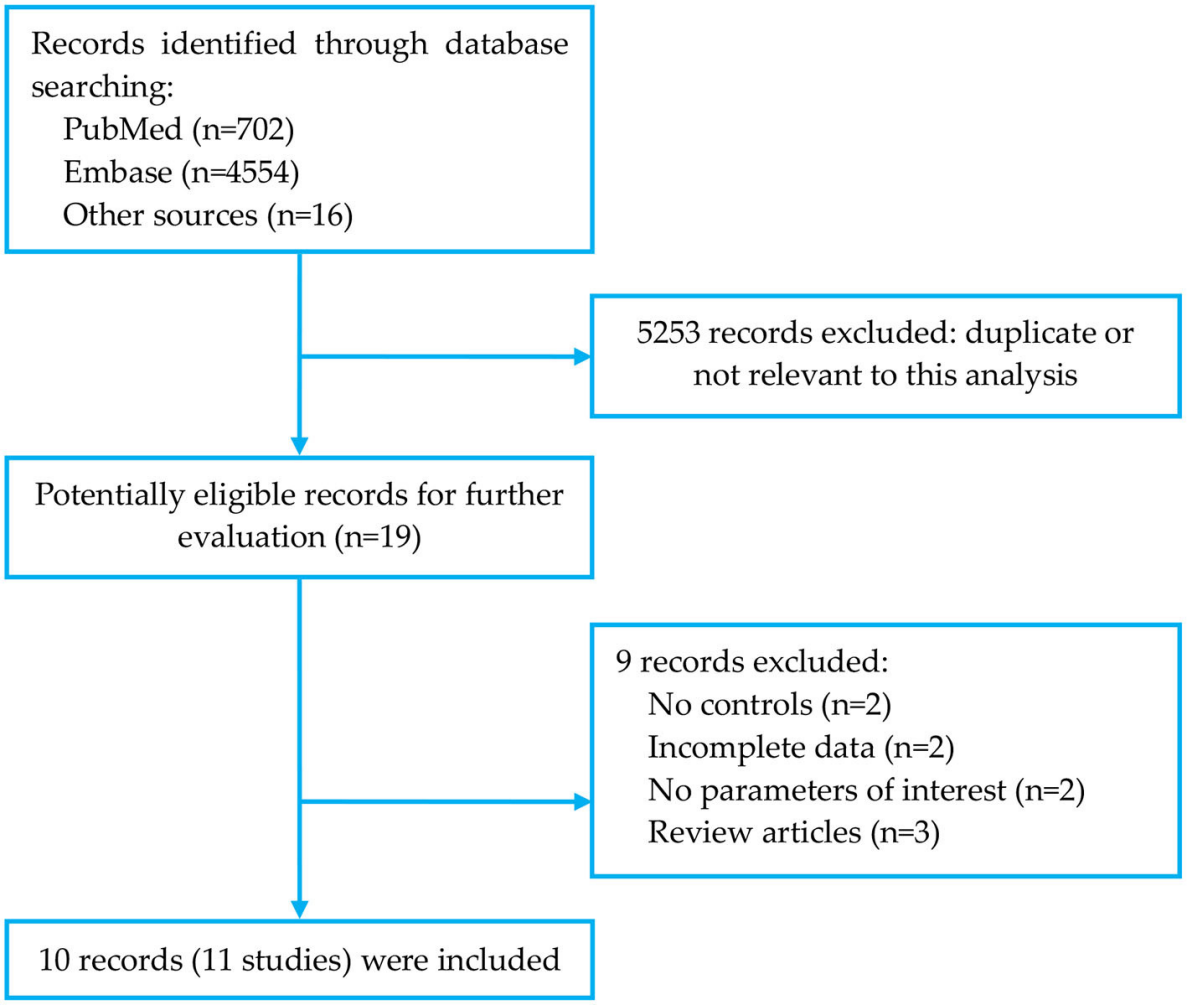
Flowchart of literature search.

The search strategy identified 5,272 records: 702 from PubMed, 4,554 from Embase, and 16 from other sources (Figure 1). After eliminating duplicated and irrelevant records, 10 recodes (11 studies) were included in the meta-analysis, with a total of 1,731 MetS cases and 11,740 controls (Table 1) (17–26). The research by Ehala-Aleksejev and Punab (19) was based on two cohorts (fertile men and male partners of infertile couples), and thus it was divided into two individual studies. All studies were cross-sectional designed. Four studies focused on infertile cohorts, two on fertile cohorts and five on the general cohort (not specified). Six studies were from Europe, three from Africa and two from Asia. In quality assessment (NOS score 0~9), the included studies had an average score of 6.73 (Table S1).

**Table 1:** Characteristics of included studies.

**References**	**Area**	**Study population**	**MetS diagnosis criteria**	**Parameters**	**MetS**		**Controls**	
					**Num**	**Levels**	**Num**	**Levels**
Saikia et al. (17)	Gauhati, India	MetS and age-matched controls (20~40y)	International Diabetes Federation (IDF) criteria in 2005	FSH (IU/L), median (range)	50	0.97 (0.76~1.1)	30	3.8 (3~4.2)
				T (ng/mL), median (range)		2.32 (1.5~4.5)		4.04 (1.98~5.98)
				InhB (pg/mL), median (range)		22.25 (14.42~36)		124.43 (88.84~198.94)
				Total sperm count (million/mL), median (range)		14 (10~22)		70 (50~78)
				Total sperm volume (mL), median (range)		3.15 (2.4~4.2)		3.45 (2.5~4.2)
				Total sperm motility (%), median (range)		69.5 (58~82)		79.5 (70~86)
				Progressive motility (%), median (range)		36 (32~45)		54 (50~59)
				Normal morphology (%), median (range)		82 (64~90)		80 (70~85)
Chen et al. (18)	Taipei, China	Participants of reproductive age (mean age 32~34y)	Harmonized criteria in 2009	Sperm concentration, mean (SD)	885	51.4 (40.21)	7,510	53.16 (40.89)
				Sperm total motility (%), mean (SD)		63.3 (18.11)		64.49 (16.17)
				Sperm progressive motility (%), mean (SD)		46.08 (18.05)		45.88 (16.68)
				Sperm normal morphology (%), mean (SD)		65.55 (17.59)		67.33 (16.41)
Ehala-Aleksejev and Punab (FM) (19)	Tartu, Estonia	Partners of pregnant women (FM) (mean age 32y)	National Cholesterol Education in Program (NCEP) criteria in 2004	Semen volume (mL), mean (95% CI)	29	4.2 (3.5~5)	209	3.7 (3.5~4)
				Sperm concentration (10^6^/mL), mean (95% CI)		55.9 (41.4~75.4)		77.7 (69.9~86.3)
				Total sperm count (10^6^), mean (95% CI)		234.9 (175.2~314.5)		289.5 (257.8~325.1)
				Motile spermatozoa (%), mean (95% CI)		51.8 (48.3~55.4)		51.5 (49.9~53.2)
				Normal morphology (%), mean (95% CI)		9.2 (7.3~11.2)		11.2 (10.5~12)
				FSH (IU/L), mean (95% CI)		4 (3.3~5.1)		4.2 (3.8~4.6)
				LH (IU/L), mean (95% CI)		4.2 (3.6~4.9)		3.7 (3.4~3.9)
				Testosterone (nmol/L), mean (95% CI)		13.5 (11.4~15.7)		17.3 (16.5~18.1)
				Estradiol (pmol/L), mean (95% CI)		120.8 (101~140.4)		124 (117.2~130.9)
Ehala-Aleksejev and Punab (MPIC) (19)	Tartu, Estonia	Male partners of infertile couples (MPIC) (mean age 33y)	National Cholesterol Education in Program (NCEP) criteria in 2004	Semen volume (mL), mean (95% CI)	471	3.6 (3.5~3.8)	2,171	3.9 (3.8~4)
				Sperm concentration (10^6^/mL), mean (95% CI)		40 (36.5~45.9)		37.8 (35.8~40)
				Total sperm count (10^6^), mean (95% CI)		142.9 (126.1~162.1)		144.3 (136.3~152.6)
				Motile spermatozoa (%), mean (95% CI)		40.9 (39.4~42.4)		41.5 (40.8~42.1)
				Normal morphology (%), mean (95% CI)		6.9 (6.4~7.4)		7 (6.7~7.2)
				FSH (IU/L), mean (95% CI)		4.3 (4~4.6)		4.3 (4.2~4.5)
				LH (IU/L), mean (95% CI)		3.3 (3.1~3.5)		3.6 (3.5~3.7)
				Testosterone (nmol/L), mean (95% CI)		13.2 (12.5~13.8)		17.4 (17.2~17.7)
				Estradiol (pmol/L), mean (95% CI)		139.6 (133.5~145.7)		130.6 (127.7~133.5)
Ventimiglia et al. (20)	Milan, Italy	Secondary infertile men (22~68y)	National Cholesterol Education in Program (NCEP) criteria in 2004	FSH (mIU/mL), mean (range)	20	9.49 (0.3~20.4)	147	6.74 (0.1~93.97)
				LH (mIU/mL), mean (range)		4.88 (0.1~10)		4.47 (0.6~32.8)
				InhB (pg/mL), mean (range)		75.3 (6~129.2)		114.6 (0.5~245.7)
				AMH (ng/mL), mean (range)		2.52 (1.3~4.4)		7.04 (0.6~19.3)
				tT (ng/mL), mean (range)		3.44 (2~6.26)		4.92 (1.75~9.73)
				E2 (pg/mL), mean (range)		35.89 (12~69)		34.91 (11~104)
				PRL (ng/mL), mean (range)		15.58 (1.22~319)		14.29 (1.08~751)
				TSH (μUI/mL), mean (range)		1.98 (0.65~5.06)		1.83 (0.01~15.58)
				Semen volume (mL), mean (range)		1.31 (0.1~5)		2.58 (0.1~10)
				Sperm concentration (10^6^/mL), mean (range)		20.08 (0~52.2)		34.53 (0~167)
				Progressive motility (%), mean (range)		18.78 (0~50)		25.28 (0~78)
				Normal morphology (%), mean (range)		1.44 (0~6)		8.01 (0~70)
Ventimiglia et al. (21)	Milan, Italy	Primary infertile men (mean age 36y)	National Cholesterol Education in Program (NCEP) criteria in 2004	FSH (mIU/mL), median (IQR)	128	5.2 (3.3~17)	1,209	5.7 (3.1~12.7)
				LH (mIU/mL), median (IQR)		4 (2.8~6.6)		4.1 (2.7~6.1)
				InhB (pg/mL), median (IQR)		40 (27.3~114.7)		85.8 (24.3~142.9)
				AMH (ng/mL), median (IQR)		4.1 (1.6~5.4)		4.7 (2.4~9.6)
				tT (ng/mL), median (IQR)		3.8 (2.7~5.3)		4.7 (3.6~6)
				E2 (pg/mL), median (IQR)		32 (24~41)		32 (25~42)
				PRL (ng/mL), median (IQR)		8.5 (3.4~18.2)		8 (3~18)
				TSH (μUI/mL), median (IQR)		1.7 (1.2~2.6)		1.6 (1.1~2.2)
				Semen volume (mL), median (IQR)		2 (0.1~3)		2 (0.1~2.5)
				Sperm concentration (10^6^/mL), median (IQR)		13.8 (2.2~40.8)		14.2 (3.8~44.1)
				Progressive motility (%), median (IQR)		25 (11~44)		25 (10~40)
				Normal morphology (%), median (IQR)		5 (0~16)		4 (0~12)
				Total sperm count, median (IQR)		25.3 (5.7~72.8)		28.7 (6.3~75.4)
Pilatz et al. (22)	Giessen, Germany	MetS and controls (30~62y)	National Cholesterol Education Program (NCEP) criteria in 2001 and International Diabetes Federation (IDF) criteria in 2009	Volume (mL), median (range)	27	2.7 (0.2~8.5)	27	3 (1~7.8)
				Sperm concentration (10^6^/mL), median (range)		52 (0.01~379)		58 (5.8~404)
				Progressive motility (%), median (range)		48 (16~64)		43 (0~72)
				Sperm morphology (normal forms, %), median (range)		4 (0~14)		5 (0~18)
Leisegang et al. (23)	Bellville and Stellenbosch, South Africa	MetS and controls (25~65y)	International Diabetes Federation (IDF) criteria in 2009	Ejaculation volume (mL), median (IQR)	32	2 (1.2~2.5)	42	2.55 (1.95~3.5)
				Sperm concentration (million/mL), median (IQR)		26.7 (15.8)		43.7 (24.6)
				Total sperm count (million), median (IQR)		48.1 (25.5~65.8)		103.6 (65.6~139.5)
				Sperm vitality (% sperm alive), median (IQR)		50 (23.2)		67.3 (15.4)
				Progressive motility (% motile), median (IQR)		20 (17.1)		29.4 (17.2)
				Total motility (% motile), median (IQR)		42.9 (19.9)		57.5 (20.8)
				MMP (% abnormal), median (IQR)		63.1 (22.2)		42.1 (25.8)
				DNA fragmentation (% abnormal), median (IQR)		26.9 (19.7)		13.9 (9.8)
Elsamanoudy et al. (24)	Mansoura, Egypt	Fertile MetS and controls (mean age 39~40y)	International Diabetes Federation (IDF) criteria in 2009	Volume (mL), mean (SD)	38	2.37 (0.67)	45	2.18 (0.54)
				Sperm concentration (10^6^/mL), mean (SD)		37.78 (9.91)		39.45 (14.2)
				Progressive motility (%), mean (SD)		43.68 (11.24)		49.67 (14.66)
				Vitality (%), mean (SD)		54.73 (16.14)		68.7 (22.04)
				Normal morphology (%), mean (SD)		22.44 (5.02)		23.53 (6.78)
				DNA fragmentation (%), mean (SD)		26.95 (9.43)		20.78 (7.15)
Leisegang et al. (25)	Western Cape, South Africa	MetS and controls (24~67y)	International Diabetes Federation (IDF) criteria in 2009	Ejaculation volume (mL), mean (SD)	24	2.3 (1.6)	26	2.7 (1)
				Sperm concentration (10^6^/mL), mean (SD)		24.6 (14.6)		43.2 (25.4)
				Total sperm count (10^6^), mean (SD)		59.3 (57.1)		122 (108.2)
				Progressive motility (%), mean (SD)		21.7 (18.3)		31 (17.6)
				Total motility (%), mean (SD)		41.8 (20.6)		54.8 (20.2)
				Vitality (%), mean (SD)		47.2 (25)		67 (16)
				Disturbed MMP (%), mean (SD)		62.4 (22.3)		40.3 (24.5)
				TUNEL-pos (%), mean (SD)		29.8 (20.4)		17.8 (12.1)
Lotti et al. (26)	Florence, Italy	Male members of infertile couples	International Diabetes Federation (IDF) criteria in 2009	FSH (IU/L), median (IQR)	27	5.6 (3.3~9.2)	324	4.8 (3~7.7)
				LH (IU/L), median (IQR)		3.9 (2.8~4.5)		3.7 (2.6~5.2)
				PRL (pmol/L), median (IQR)		282 (234~489)		294 (226~435)
				TSH (mIU/L), median (IQR)		1.84 (1.13~2.26)		1.51 (1.08~2.09)
				Total testosterone (nmol/L), mean (SD)		13.8 (6.5)		16.7 (6.2)
				Semen volume (mL), median (IQR)		2.8 (1.3~3.8)		3 (2~4.2)
				Sperm concentration (10^6^/mL), median (IQR)		16.1 (3.9~49.5)		13 (1.6~46)
				Sperm progressive motility (%), mean (SD)		39.3 (16.9)		36.2 (20.7)
				Sperm morphology (% normal), median (IQR)		4 (2~6.3)		5 (2~10)

*MetS, metabolic syndrome; FSH, follicle-stimulating hormone; T, testosterone; tT, total testosterone; InhB, inhibin B; LH, luteinizing hormone; AMH, anti-Müllerian hormone; TSH, thyroid-stimulating hormone; E2, estradiol; PRL, prolactin; MMP, mitochondrial membrane potential; IQR, inter-quartile range; SD, standard error; Num, number*.

### MetS and Semen Quality

**Figure 2:**
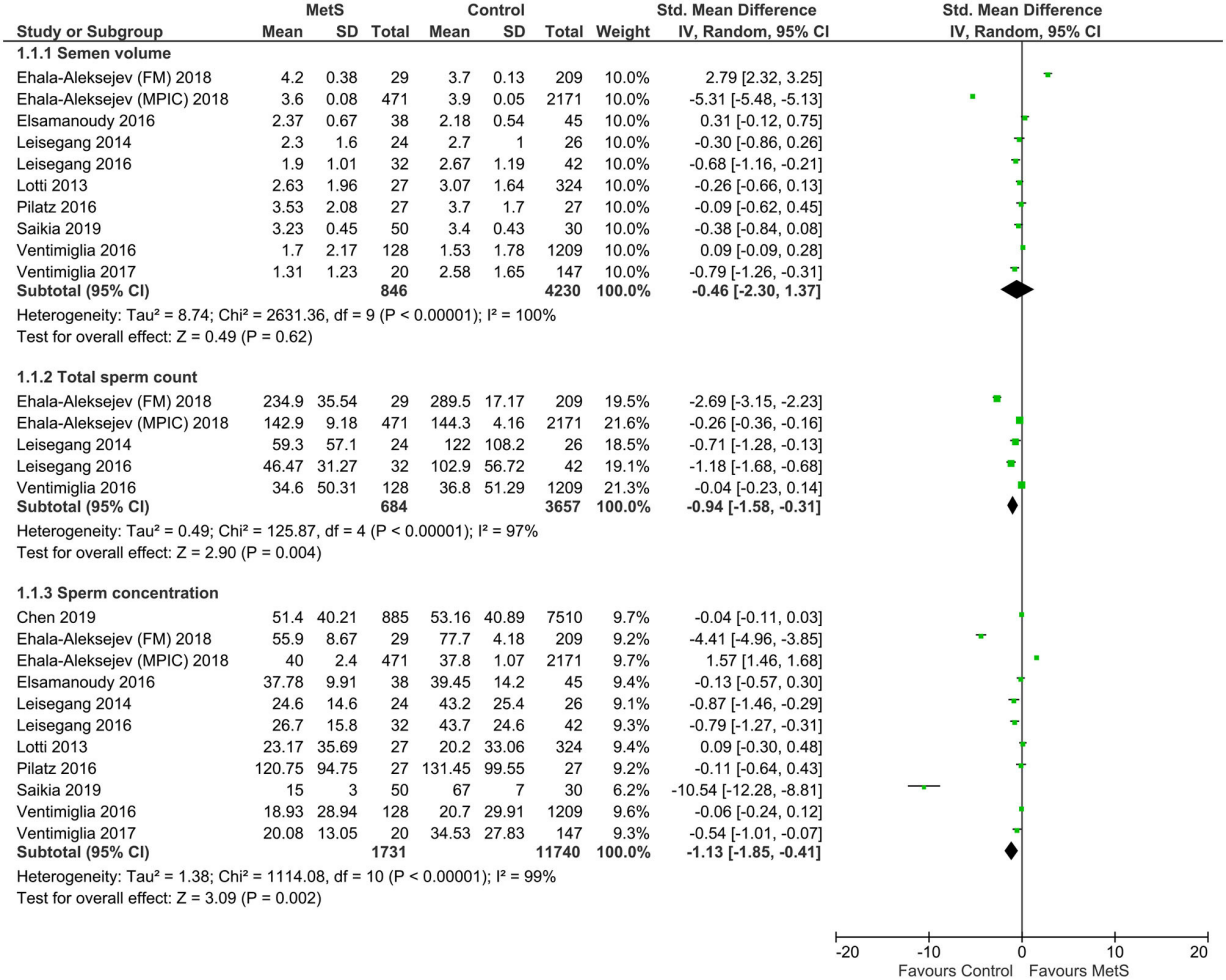
Meta-analysis of the effects of metabolic syndrome on semen volume, sperm total count, and sperm concentration.

**Figure 3:**
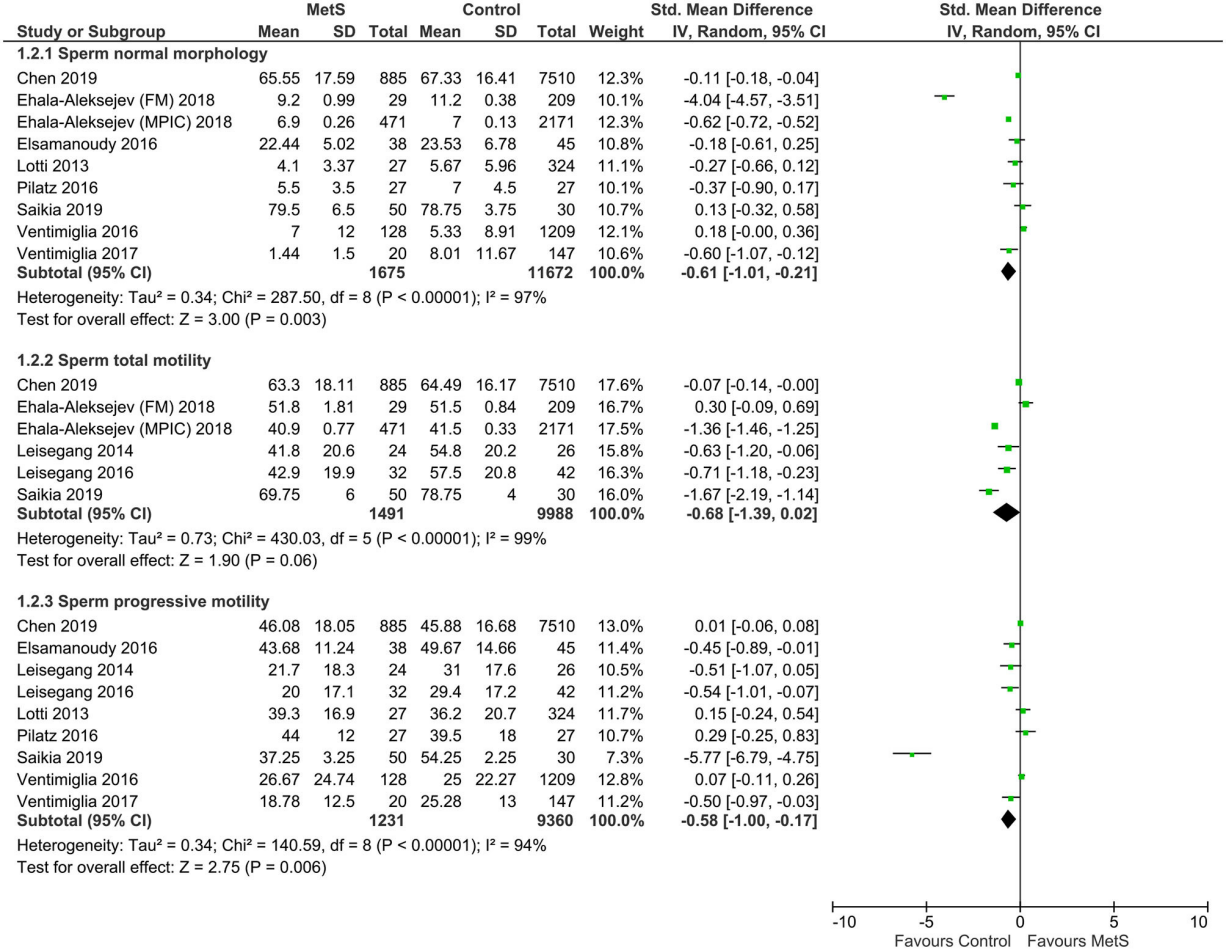
Meta-analysis of the effects of metabolic syndrome on sperm normal morphology, sperm total motility, and sperm progressive motility.

**Figure 4:**
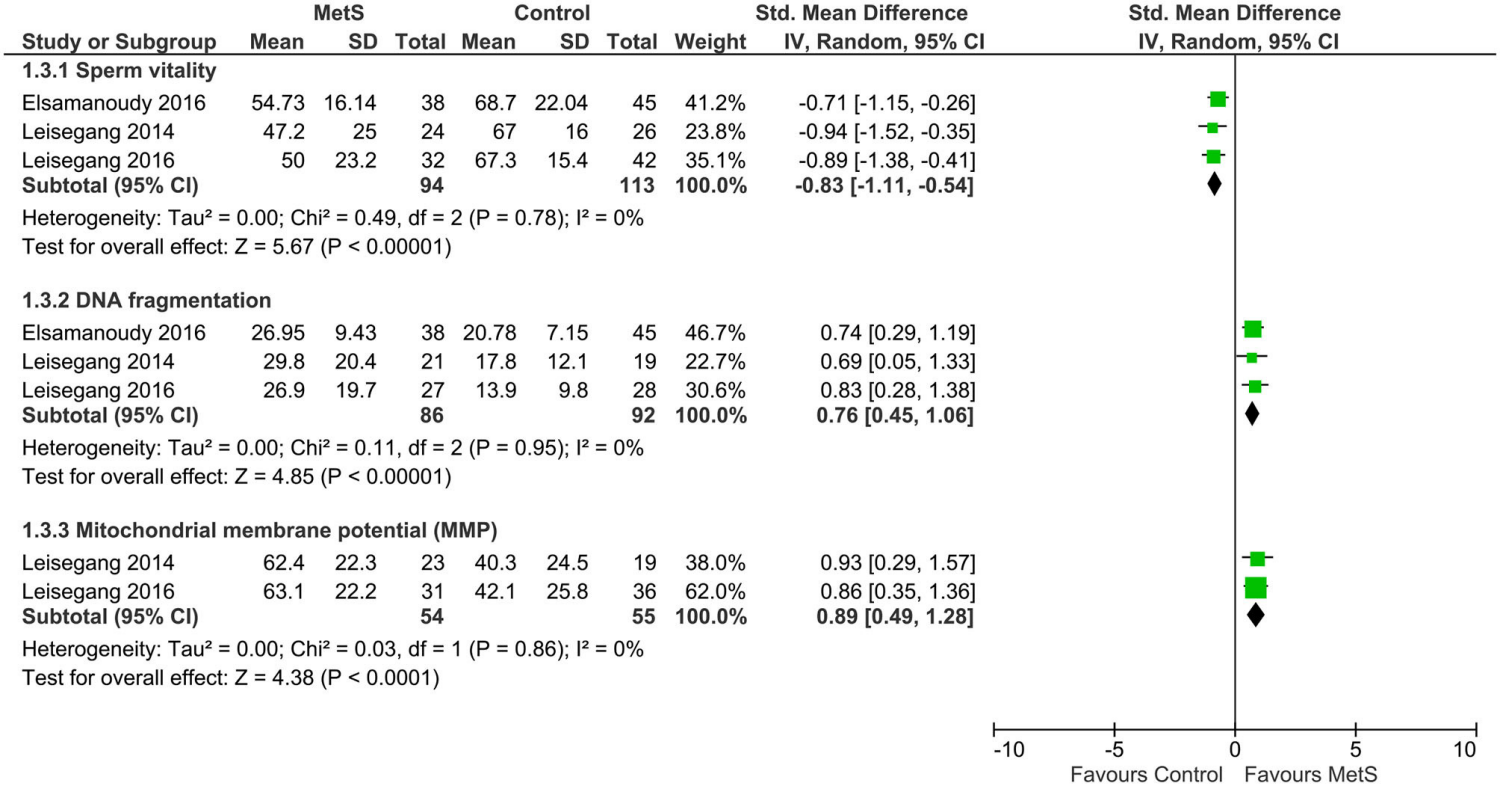
Meta-analysis of the effects of metabolic syndrome on sperm vitality, DNA fragmentation, and mitochondrial membrane potential (MMP).

Compared with the controls, MetS cases had a statistically significant decrease of sperm total count (SMD: −0.96, 95% CI: −1.58 to −0.31; *I*^2^ = 97%, *n* = 5), sperm concentration (SMD: −1.13, 95% CI: −1.85 to −0.41; *I*^2^ = 99%, *n* = 11), sperm normal morphology (SMD: −0.61, 95% CI: −1.01 to −0.21; *I*^2^ = 97%, *n* = 9), sperm progressive motility (SMD: −0.58, 95% CI: −1.00 to −0.17; *I*^2^ = 94%, *n* = 9), and sperm vitality (SMD: −0.83, 95% CI: −1.11 to −0.54; *I*^2^ = 0%, *n* = 3) (Figures 2–4). There was found a weak decrease of semen volume (SMD: −0.46, 95% CI: −2.30 to 1.37; *I*^2^ = 100%, *n* = 10) and sperm total motility in MetS cases (SMD: −0.68, 95% CI: −1.39 to 0.02; *I*^2^ = 99%, *n* = 6). Furthermore, MetS cases had a statistically significant increase of sperm DNA fragmentation (SMD: 0.76, 95% CI: 0.45 to 1.06; *I*^2^ = 0%, *n* = 3) and MMP (SMD: 0.89, 95% CI: 0.49 to 1.28; *I*^2^ = 0%, *n* = 2).

### MetS and Circulating Sex Hormones

**Figure 5:**
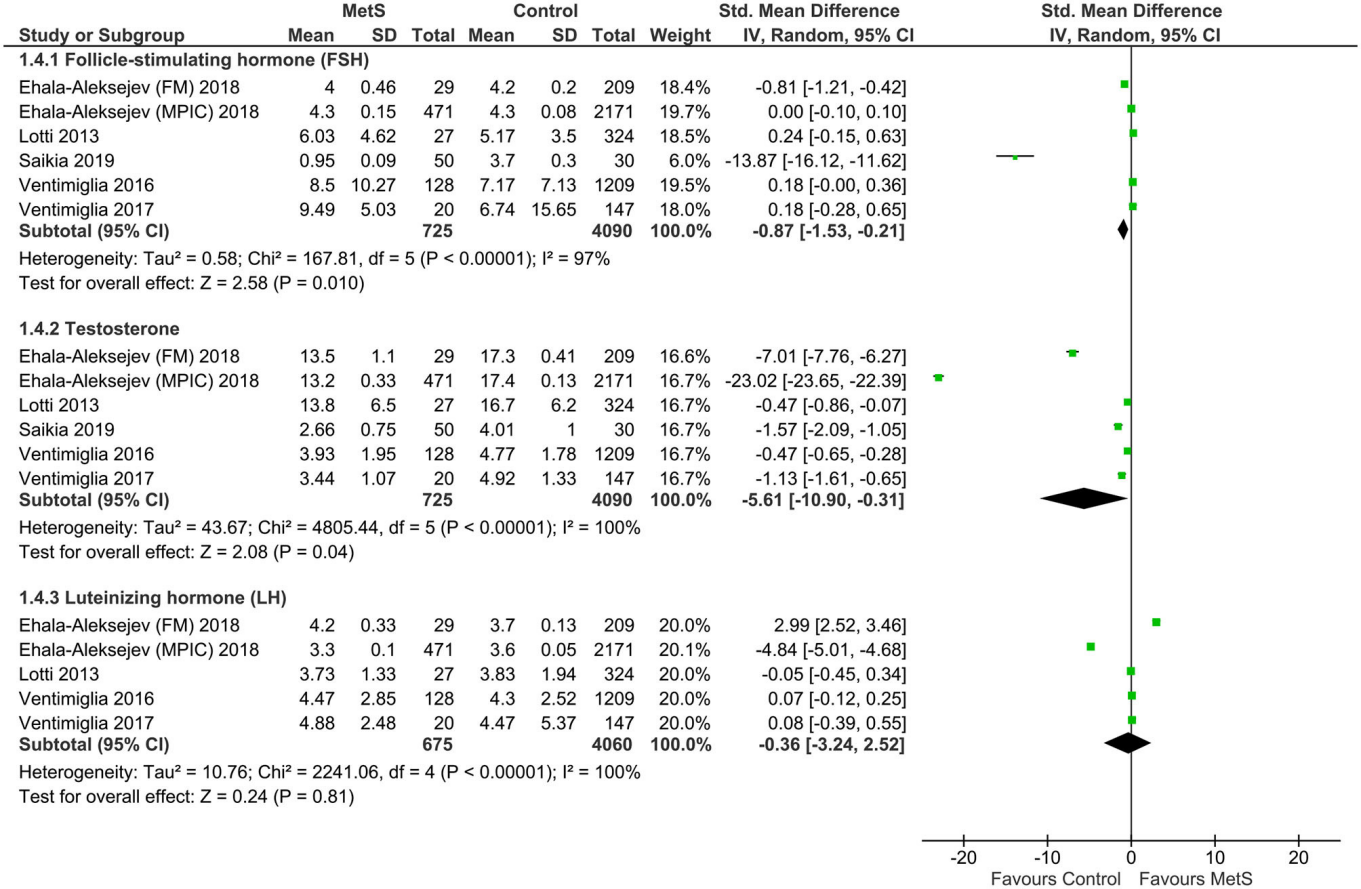
Meta-analysis of the effects of metabolic syndrome on circulating follicle-stimulating hormone (FSH), testosterone, and luteinizing hormone (LH).

**Figure 6:**
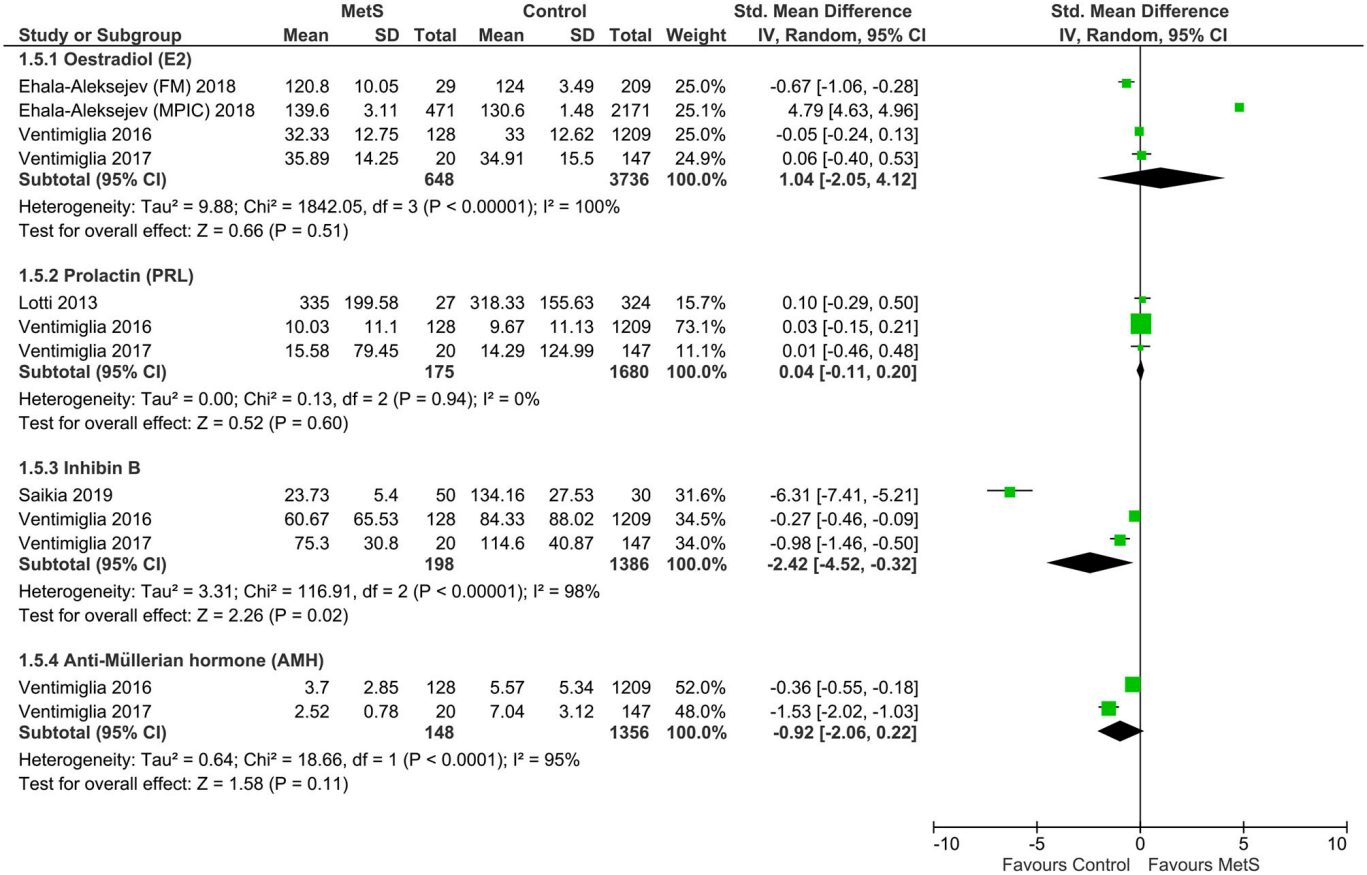
Meta-analysis of the effects of metabolic syndrome on estradiol, inhibin B, and anti-Müllerian hormone (AMH).

Compared with the controls, MetS cases had a statistically significant decrease of follicle-stimulating hormone (FSH) (SMD: −0.87, 95% CI: −1.53 to −0.21; *I*^2^ = 97%, *n* = 6**)**, testosterone (SMD: −5.61, 95% CI: −10.90 to −0.31; *I*^2^ = 100%, *n* = 6**)**, and inhibin B (SMD: −2.42, 95% CI: −4.52 to −0.32; *I*^2^ = 0%, *n* = 3) (Figures 5, 6). There was found a weak decrease of luteinizing hormone (LH) (SMD: −0.36, 95% CI: −3.24 to 2.52; *I*^2^ = 100%, *n* = 5) and anti-Müllerian hormone (AMH) (SMD: −0.92, 95% CI: −2.06 to 0.22; *I*^2^ = 95%, *n* = 2), and a weak increase of estradiol (SMD: 1.04, 95% CI: −2.05 to 4.12; *I*^2^ = 100%, *n* = 4) in MetS cases.

### Sensitivity Analyses

Sensitivity analysis showed that the results were robust in semen parameters. In the meta-analysis of circulating FSH, the result showed no statistical difference when omitting the study by Saikia et al. (17) (Table S2). In the meta-analysis of testosterone, the result also showed no statistical difference when omitting the study by Ehala-Aleksejev and Punab (19) (FM), Lotti et al. (26), or Ventimiglia et al. (21).

### Subgroup Analyses

Subgroup analyses were conducted on the study cohort, ethnicity and study area. Compared with the infertile cohort, MetS cases in the fertile cohort had a significantly higher incidence of a decrease in sperm total count and sperm progressive motility, FSH, testosterone and estradiol, and an increase in LH (Table 2). Comparably, MetS cases in the general cohort also had a significantly higher incidence of the decrease in semen volume, sperm total count, sperm concentration, sperm normal morphology, sperm progressive motility, FSH, testosterone, and inhibin B. Generally, MetS had the most impact on semen quality and circulating sex hormones of the general cohort, moderate impact on fertile cohort, and the least impact on the infertile cohort.

**Table 2:** Subgroup analysis of the effects of metabolic syndrome on semen quality and circulating sex hormones according to the study cohort.

**Variables**	**Fertile cohort**		**Infertile cohort**		**Not specified**	
	**SMD (95% CI)**	**No**.	**SMD (95% CI)**	**No**.	**SMD (95% CI)**	**No**.
Semen volume	1.55 (−0.88 to 3.97)	2	−1.57 (−4.81 to 1.68)	4	−0.38 (−0.63 to −0.13)	4
Sperm total count	−2.69 (−3.15 to −2.23)	1	−0.16 (−0.37 to 0.04)	2	−0.96 (−1.42 to −0.50)	2
Sperm concentration	−2.27 (−6.45 to 1.92)	2	0.27 (−0.83 to 1.38)	4	−1.99 (−3.23 to −0.75)	5
Sperm normal morphology	−2.11 (−5.89 to 1.68)	2	−0.32 (−0.81 to 0.17)	4	−0.11 (−0.17 to −0.04)	3
Sperm total motility	0.30 (−0.09 to 0.69)	1	−1.36 (−1.46 to −1.25)	1	−0.75 (−1.47 to −0.02)	4
Sperm progressive motility	−0.45 (−0.89 to −0.01)	1	−0.05 (−0.37 to 0.28)	3	−1.18 (−2.26 to −0.11)	5
Sperm vitality	−0.71 (−1.15 to −0.26)	1	–	–	−0.91 (−1.28 to −0.54)	2
DNA fragmentation	0.74 (0.29 to 1.19)	1	–	–	0.77 (0.35 to 1.19)	2
Mitochondrial membrane potential (MMP)	–	–	–	–	0.89 (0.49 to 1.28)	2
Follicle-stimulating hormone (FSH)	−0.81 (−1.21 to −0.42)	1	0.08 (−0.04 to 0.21)	4	−13.87 (−16.12 to −11.62)	1
Testosterone	−7.01 (−7.76 to −6.27)	1	−6.27 (−13.71 to 1.18)	4	−1.57 (−2.09 to −1.05)	1
Luteinizing hormone (LH)	2.99 (2.52 to 3.46)	1	−1.19 (−4.24 to 1.86)	4	–	–
estradiol	−0.67 (−1.06 to −0.28)	1	1.60 (−2.07 to 5.28)	3	–	–
Prolactin	–	–	0.04 (−0.11 to 0.20)	3	–	–
Inhibin B	–	–	−0.59 (−1.28 to 0.10)	2	−6.31 (−7.41 to −5.21)	1
Anti-Müllerian hormone (AMH)	–	–	−0.92 (−2.06 to 0.22)	2	–	–

*SMD, standardized mean differences; CI, confidence interval; No., number of included studies*.

Compared with the Caucasian cohort or the cohort from developed area, MetS cases from the non-Caucasian cohort or the cohort from developing area had a significantly higher incidence of the decrease in sperm concentration, sperm total motility, sperm progressive motility, FSH, and inhibin B (Table 3). MetS tended to have more impact on the individuals from the non-Caucasian cohort or the cohort from developing area.

**Table 3:** Subgroup analysis of the effects of metabolic syndrome on semen quality and circulating sex hormones according to the ethnicity and study area.

**Variables**	**Caucasian cohort/developed area**		**Non-Caucasian cohort/developing area**	
	**SMD (95% CI)**	**No**.	**SMD (95% CI)**	**No**.
Semen volume	−0.60 (−3.28 to 2.09)	6	−0.25 (−0.69 to 0.18)	4
Sperm total count	−0.95 (−1.79 to −0.11)	3	−0.96 (−1.42 to −0.50)	2
Sperm concentration	−0.56 (−1.82 to 0.70)	6	−1.94 (−3.10 to −0.78)	5
Sperm normal morphology	−0.93 (−1.66 to −0.19)	6	−0.10 (−0.17 to −0.04)	3
Sperm total motility	−0.54 (−2.16 to 1.08)	2	−0.75 (−1.47 to −0.02)	4
Sperm progressive motility	0.02 (−0.25 to 0.28)	4	−1.32 (−2.34 to −0.30)	5
Sperm vitality	–	–	−0.83 (−1.11 to −0.54)	3
DNA fragmentation	–	–	0.76 (0.45 to 1.06)	3
Mitochondrial membrane potential (MMP)	–	–	0.89 (0.49 to 1.28)	2
Follicle-stimulating hormone (FSH)	−0.03 (−0.29 to 0.23)	5	−13.87 (−16.12 to −11.62)	1
Testosterone	−5.61 (−10.90 to −0.31)	5	−6.41 (−12.81 to −0.02)	1
Luteinizing hormone (LH)	−0.36 (−3.24 to 2.52)	5	–	–
estradiol	1.04 (−2.05 to 4.12)	4	–	–
Prolactin	0.04 (−0.11 to 0.20)	3	–	–
Inhibin B	−0.59 (−1.28 to 0.10)	2	−6.31 (−7.41 to −5.21)	1
Anti-Müllerian hormone (AMH)	−0.92 (−2.06 to 0.22)	2	–	–

*SMD, standardized mean differences; CI, confidence interval; No., number of included studies*.

### Publication Bias

Egger's test was conducted on the indicators with more than four included studies. Finally, we detected no significant publication bias in semen volume (*P* = 0.122), sperm total count (*P* = 0.200), sperm concentration (*P* = 0.185), sperm normal morphology (*P* = 0.400), sperm total motility (*P* = 0.659), sperm progressive motility (*P* = 0.120), circulating FSH (*P* = 0.199), testosterone (*P* = 0.215), or LH (*P* = 0.293) (Figures S1–S3).

## Discussion

According to the World Health Organization (WHO), infertility had an incidence of 8~12% in childbearing couples worldwide, among which male infertility accounted for 40~50% (27). Along with the modernized lifestyles of recent decades, metabolic disorders were increasingly prevalent, while semen quality was gradually decreasing (28). Thus, as a collection of metabolic disorders characterized by abdominal obesity, dyslipidemia, hypertension and insulin resistance, MetS was thought to be involved in the pathogenesis of male infertility (28).

The mechanism has been not clarified, and currently many researchers indicate a central role of insulin resistance in the pathogenesis. Abnormal blood glucose could cause the impairment of multiple organs, including erectile and ejaculation disorders. In the meta-analysis by Pergialiotis et al. (29) the infertile male with diabetes had a decrease in seminal volume and motile cells and an increase in FSH. Moreover, diabetes patients in fertile age had a higher prevalence of male accessory gland inflammations/infections, as well as a higher failure rate of vitro fertilization (30, 31). Antidiabetic agents could not only control blood glucose levels but also improve semen quality and testosterone levels (32, 33). Besides, individuals with obesity, dyslipidemia or hypertension were also reported with a decrease in semen quality and changes in sex hormones. These might contribute to concomitant oxidative stress and inflammation, and impaired seminal antioxidant capacity (34, 35).

In this meta-analysis, we found a decrease of sperm total count, sperm concentration, sperm normal morphology, sperm progressive motility, and sperm vitality and an increase of sperm DNA fragmentation and MMP, while no significant difference was found in semen volume and sperm total motility. Generally, MetS had a negative impact on the semen quality, just like diabetes and obesity (10, 36). On the other hand, MetS cases had a decrease of FSH, testosterone and inhibin B, while no significant difference was found in LH, estradiol, prolactin, and AMH. Previous studies reported a decrease of inhibin B and an increase of FSH in infertile males (37). However, our meta-analysis indicated a similar change trend of FSH and inhibin B in MetS. This might contribute to the heterogeneity between studies, especially from the study by Saikia et al. (17). Second, LH and estradiol were usually increased in infertile males, but our meta-analysis found no obvious difference in MetS. Apart from the heterogeneity, this might also contribute to the complexity of MetS as a syndrome. For example, as one of the characteristics of MetS, obese males could have an increase of testosterone, LH and FSH after bariatric surgery (38). In general, MetS had a greater impact on semen quality than sex hormones, which might contribute to the direct impairment caused by MetS.

Sensitivity analysis indicated a relative stability for semen parameters, while FSH and testosterone turned statistically insignificant when omitting certain studies. MetS seemed to have more significant and stable effects on semen quality than sex hormones, which was consistent with our previous analysis. Second, for almost all the outcomes, the exclusion of a single specific study dramatically decreased the effect size, especially like Ehala-Aleksejev and Punab (19) (FM) in sperm concentration, sperm normal morphology and testosterone, Saikia et al. (17) in sperm progressive motility and FSH, and Lotti et al. (26) in testosterone. These studies were limited in the sample size of MetS cases ranging from 27 to 50. Small sample size could increase the risk of sampling error, and thus lead to within-study and between-study heterogeneity and the expansion of synthetic effect size. Besides, the meta-analysis of continuous data usually showed a higher heterogeneity than categorical data, just like the recent study of “Cardio-metabolic risk factors among young infertile women: a systematic review and meta-analysis” (35).

The subgroup analyses suggested more effects of MetS on the individuals from the fertile cohort, non-Caucasian cohort, or the cohort from developing area. This might contribute to less impact of MetS on the impaired reproductivity, and MetS had a stronger influence on the reproductivity of healthy individuals. Moreover, this was also consistent with the high incidence of male infertility in the Asian cohort and developing countries (27).

Although this was the first meta-analysis to evaluate the effects of MetS on both semen quality and circulating sex hormones in men, several limitations in this study should be also considered. First, not all included studies had a large sample size. Second, all included studies were cross-sectionally designed, and prospective studies were needed to confirm our findings. Third, the protocol of our meta-analysis was not registered in the PROSPERO database. Fourth, obvious heterogeneity between studies was observed, although we conducted both sensitivity analysis and subgroup analysis to evaluate the stability of the results. We expected large-scale prospective designed studies in the future to overcome these limitations.

In conclusion, this meta-analysis demonstrated the effects of MetS on almost all the semen parameters and part of the circulating sex hormones, and MetS tended to be a risk factor for male infertility. Further larger-scale prospective designed studies were needed to confirm our findings.

## Data Availability Statement

The raw data supporting the conclusions of this article will be made available by the authors, without undue reservation.

## Author Contributions

LZ and AP conceived the manuscript, performed literature search, drafted and wrote the manuscript, contributed to manuscript revision during peer review process, and contributed to manuscript revision, read, and approved the submitted version. AP critically revised the first original draft and any other version of the manuscript before and after peer review process, and provided significant content contribution and English language support.

## Conflict of Interest

The authors declare that the research was conducted in the absence of any commercial or financial relationships that could be construed as a potential conflict of interest.
