# Supplementary material for: Effects of Metabolic Syndrome on Semen Quality and Circulating Sex Hormones: A Systematic Review and Meta-Analysis
Source: Front Endocrinol (Lausanne). 2020 Aug 11;11:428. doi: 10.3389/fendo.2020.00428 (PMC7431460; doi:10.3389/fendo.2020.00428)
Supplement: Supplementary file 1 [file Data_Sheet_1.docx]

**Table S1.** Methodological qualtiy of include studies in the meta-analysis.

| **First author, year** | **Adequate definition of cases** | **Representativeness of cases** | **Selection of control subjects** | **Definition of control subjects** | **Control for important factors** | **Exposure assessment** | **Same method of ascertainment for all subjects** | **Non-response rate** | **Using an adjusted model** | **NOS score** |
| --- | --- | --- | --- | --- | --- | --- | --- | --- | --- | --- |
| Saikia 2019 | ☆ | ☆ | ☆ | ☆ | ☆ | - | ☆ | ☆ | - | 7 |
| Chen 2019 | ☆ | ☆ | ☆ | ☆ | ☆ | - | ☆ | ☆ | - | 7 |
| Ehala-Aleksejev (FM) 2018 | ☆ | ☆ | ☆ | ☆ | ☆ | - | ☆ | ☆ | - | 7 |
| Ehala-Aleksejev (MPIC) 2018 | ☆ | ☆ | ☆ | ☆ | ☆ | - | ☆ | ☆ | - | 7 |
| Ventimiglia 2017 | ☆ | ☆ | ☆ | ☆ | ☆ | - | ☆ | ☆ | - | 7 |
| Ventimiglia 2016 | ☆ | ☆ | ☆ | ☆ | ☆ | - | ☆ | ☆ | - | 7 |
| Pilatz 2016 | ☆ | ☆ | ☆ | ☆ | - | - | ☆ | ☆ | - | 6 |
| Leisegang 2016 | ☆ | ☆ | ☆ | ☆ | - | - | ☆ | ☆ | - | 6 |
| Elsamanoudy 2016 | ☆ | ☆ | ☆ | ☆ | ☆ | - | ☆ | ☆ | - | 7 |
| Leisegang 2014 | ☆ | ☆ | ☆ | ☆ | - | - | ☆ | ☆ | - | 6 |
| Lotti 2013 | ☆ | ☆ | ☆ | ☆ | ☆ | - | ☆ | ☆ | - | 7 |

NOS, the Newcastle-Ottawa Scale.

**Table S2.** Sensitivity analysis in the meta-analysis.

| **Study omitted** | **SMD (95% CI)** |
| --- | --- |
| **Semen volume** |  |
| Ehala-Aleksejev (FM) 2018 | -0.82 (-2.68 to 1.03) |
| Ehala-Aleksejev (MPIC) 2018 | 0.08 (-0.52 to 0.68) |
| Elsamanoudy 2016 | -0.55 (-2.54 to 1.44) |
| Leisegang 2014 | -0.48 (-2.46 to 1.49) |
| Leisegang 2016 | -0.44 (-2.44 to 1.56) |
| Lotti 2013 | -0.49 (-2.50 to 1.53) |
| Pilatz 2016 | -0.51 (-2.48 to 1.47) |
| Saikia 2019 | -0.47 (-2.47 to 1.52) |
| Ventimiglia 2016 | -0.53 (-2.66 to 1.60) |
| Ventimiglia 2017 | -0.43 (-2.43 to 1.57) |
| **Combined** | **-0.46 (-2.30 to 1.37)** |
| **Total sperm count** |  |
| Ehala-Aleksejev (FM) 2018 | -0.44 (-0.76 to -0.12) |
| Ehala-Aleksejev (MPIC) 2018 | -1.15 (-2.40 to -0.11) |
| Leisegang 2014 | -1.00 (-1.73 to -0.27) |
| Leisegang 2016 | -1.20 (-2.35 to -0.06) |
| Ventimiglia 2016 | -0.89 (-1.60 to -0.18) |
| **Combined** | **-0.94 (-1.58 to -0.31)** |
| **Sperm concentration** |  |
| Chen 2019 | -1.38 (-2.41 to -0.34) |
| Ehala-Aleksejev (FM) 2018 | -0.74 (-1.40 to -0.07) |
| Ehala-Aleksejev (MPIC) 2018 | -1.33 (-1.97 to -0.70) |
| Elsamanoudy 2016 | -1.24 (-2.01 to -0.48) |
| Leisegang 2014 | -1.16 (-1.91 to -0.40) |
| Leisegang 2016 | -1.17 (-1.93 to -0.41) |
| Lotti 2013 | -1.27 (-2.04 to -0.50) |
| Pilatz 2016 | -1.24 (-2.00 to -0.48) |
| Saikia 2019 | -0.51 (-1.20 to -0.18) |
| Ventimiglia 2016 | -1.29 (-2.12 to -0.46) |
| Ventimiglia 2017 | -1.20 (-1.96 to -0.44) |
| **Combined** | **-1.13 (-1.85 to -0.41)** |
| **Sperm normal morphology** |  |
| Chen 2019 | -0.70 (-1.29 to -0.11) |
| Ehala-Aleksejev (FM) 2018 | -0.22 (-0.48 to -0.04) |
| Ehala-Aleksejev (MPIC) 2018 | -0.63 (-1.17 to -0.09) |
| Elsamanoudy 2016 | -0.67 (-1.10 to -0.24) |
| Lotti 2013 | -0.66 (-1.09 to -0.22) |
| Pilatz 2016 | -0.64 (-1.07 to -0.21) |
| Saikia 2019 | -0.70 (-1.13 to -0.27) |
| Ventimiglia 2016 | -0.72 (-1.17 to -0.27) |
| Ventimiglia 2017 | -0.62 (-1.04 to -0.19) |
| **Combined** | **-0.61 (-1.01 to -0.21)** |
| **Sperm total motility** |  |
| Chen 2019 | -0.81 (-1.52 to 0.11) |
| Ehala-Aleksejev (FM) 2018 | -0.88 (-1.66 to 0.10) |
| Ehala-Aleksejev (MPIC) 2018 | -0.52 (-1.07 to 0.02) |
| Leisegang 2014 | -0.69 (-1.47 to 0.08) |
| Leisegang 2016 | -0.68 (-1.46 to 0.11) |
| Saikia 2019 | -0.50 (-1.26 to 0.27) |
| **Combined** | **-0.68 (-1.39 to 0.02)** |
| **Sperm progressive motility** |  |
| Chen 2019 | -0.78 (-1.43 to -0.12) |
| Elsamanoudy 2016 | -0.61 (-1.07 to -0.16) |
| Leisegang 2014 | -0.60 (-1.05 to -0.15) |
| Leisegang 2016 | -0.60 (-1.05 to -0.15) |
| Lotti 2013 | -0.70 (-1.16 to -0.23) |
| Pilatz 2016 | -0.70 (-1.15 to -0.25) |
| Saikia 2019 | -0.12 (-0.30 to -0.06) |
| Ventimiglia 2016 | -0.76 (-1.35 to -0.17) |
| Ventimiglia 2017 | -0.60 (-1.05 to -0.15) |
| **Combined** | **-0.58 (-1.00 to -0.17)** |
| **Sperm vitality** |  |
| Elsamanoudy 2016 | -0.91 (-1.28 to -0.54) |
| Leisegang 2014 | -0.79 (-1.12 to -0.47) |
| Leisegang 2016 | -0.79 (-1.15 to -0.44) |
| **Combined** | **-0.83 (-1.11 to -0.54)** |
| **DNA fragmentation** |  |
| Elsamanoudy 2016 | 0.77 (0.35 to 1.19) |
| Leisegang 2014 | 0.77 (0.43 to 1.12) |
| Leisegang 2016 | 0.72 (0.36 to 1.09) |
| **Combined** | **0.76 (0.45 to 1.06)** |
| **MMP** |  |
| Leisegang 2014 | 0.86 (0.35 to 1.36) |
| Leisegang 2016 | 0.93 (0.29 to 1.57) |
| **Combined** | **0.89 (0.49 to 1.28)** |
| **Circulating FSH** |  |
| Ehala-Aleksejev (FM) 2018 | -0.91 (-1.65 to -0.16) |
| Ehala-Aleksejev (MPIC) 2018 | -1.75 (-2.93 to -0.58) |
| Lotti 2013 | -1.21 (-1.99 to -0.43) |
| Saikia 2019 | -0.03 (-0.29 to 0.23) |
| Ventimiglia 2016 | -1.66 (-2.72 to -0.59) |
| Ventimiglia 2017 | -1.17 (-1.93 to -0.40) |
| **Combined** | **-0.87 (-1.53 to -0.21)** |
| **Circulating testosterone** |  |
| Ehala-Aleksejev (FM) 2018 | -5.33 (-11.28 to 0.63) |
| Ehala-Aleksejev (MPIC) 2018 | -2.10 (-3.63 to -0.56) |
| Lotti 2013 | -6.64 (-13.50 to 0.23) |
| Saikia 2019 | -6.41 (-12.81 to -0.02) |
| Ventimiglia 2016 | -6.64 (-14.18 to 0.91) |
| Ventimiglia 2017 | -6.50 (-12.99 to -0.02) |
| **Combined** | **-5.61 (-10.90 to -0.31)** |
| **Circulating LH** |  |
| Ehala-Aleksejev (FM) 2018 | -1.19 (-4.24 to 1.86) |
| Ehala-Aleksejev (MPIC) 2018 | 0.76 (-0.42 to 1.95) |
| Lotti 2013 | -0.43 (-3.85 to 2.99) |
| Ventimiglia 2016 | -0.46 (-4.31 to 3.39) |
| Ventimiglia 2017 | -0.46 (-3.82 to 2.89) |
| **Combined** | **-0.36 (-3.24 to 2.52)** |
| **Circulating oestradiol** |  |
| Ehala-Aleksejev (FM) 2018 | 1.60 (-2.07 to 5.28) |
| Ehala-Aleksejev (MPIC) 2018 | -0.21 (-0.62 to 0.19) |
| Ventimiglia 2016 | 1.40 (-2.60 to 5.40) |
| Ventimiglia 2017 | 1.36 (-2.36 to 5.08) |
| **Combined** | **1.04 (-2.05 to 4.12)** |
| **Circulating prolactin** |  |
| Lotti 2013 | 0.03 (-0.14 to 0.20) |
| Ventimiglia 2016 | 0.07 (-0.23 to 0.37) |
| Ventimiglia 2017 | 0.05 (-0.12 to 0.21) |
| **Combined** | **0.04 (-0.11 to 0.20)** |
| **Circulating inhibin B** |  |
| Saikia 2019 | -0.59 (-1.28 to 0.10) |
| Ventimiglia 2016 | -3.62 (-8.85 to 1.60) |
| Ventimiglia 2017 | -3.27 (-9.19 to 2.65) |
| **Combined** | **-2.42 (-4.52 to -0.32)** |
| **Circulating AMH** |  |
| Ventimiglia 2016 | -0.36 (-0.55 to -0.18) |
| Ventimiglia 2017 | -1.53 (-2.02 to -1.03) |
| **Combined** | **-0.92 (-2.06 to 0.22)** |

SMD, standardized mean differences; CI, confidence interval.

**Figure S1.** Funnel plot for publication bias in the meta-analysis of the effects of metabolic syndrome on semen volume, sperm total count and sperm concentration.

**Figure S2.** Funnel plot for publication bias in the meta-analysis of the effects of metabolic syndrome on sperm normal morphology, sperm total motility and sperm progressive motility.

**Figure S3.** Funnel plot for publication bias in the meta-analysis of the effects of metabolic syndrome on circulating follicle-stimulating hormone (FSH), testosterone and luteinizing hormone (LH).
